# White matter deficits in schizophrenia are global and don’t progress with age

**DOI:** 10.1177/0004867417700729

**Published:** 2017-04-06

**Authors:** Richard A Kanaan, Marco M Picchioni, Colm McDonald, Sukhwinder S Shergill, Philip K McGuire

**Affiliations:** 1 Department of Psychiatry, Austin Health, The University of Melbourne, Heidelberg, VIC, Australia; 2 Department of Psychosis Studies, Institute of Psychiatry, Psychology & Neuroscience, King’s College London, London, UK; 3 St Andrew’s Academic Department and King’s College London, Northampton, UK; 4 National University of Ireland (NUI), Galway, Ireland

**Keywords:** Diffusion tensor imaging, illness duration, antipsychotic medication, TBSS, DTI

## Abstract

**Introduction:**

Diffusion tensor imaging has revealed differences in all examined white matter tracts in schizophrenia, with a range of explanations for why this may be. The distribution and timing of differences may help explain their origin; however, results are usually dependent on the analytical method. We therefore sought to examine the extent of differences and their relationship with age using two different methods.

**Methods:**

A combined voxel-based whole-brain study and a tract-based spatial-statistics study of 104 patients with schizophrenia and 200 matched healthy controls, aged between 17 and 63 years.

**Results:**

Fractional anisotropy was reduced throughout the brain in both analyses. The relationship of fractional anisotropy with age differed between patients and controls, with controls showing the gentle fractional anisotropy decline widely noted but patients showing an essentially flat relationship: younger patients had lower fractional anisotropy than controls, but the difference disappeared with age. Mean diffusivity was widely increased in patients.

**Conclusion:**

Reduction in fractional anisotropy and increase in mean diffusivity would be consistent with global disruption in myelination; the relationship with age would suggest this is present already at the onset of their illness, but does not progress.

## Introduction

Schizophrenia is increasingly recognised as a disorder of connectivity (Fornito et al., 2012; Friston, 2002; Stephan et al., 2006), with the structural basis of that disconnection in white matter commonly examined with Diffusion Tensor Magnetic Resonance Imaging (DTI) (Basser et al., 1994). DTI can be used to generate scalar measures, notably mean diffusivity (MD) and fractional anisotropy (FA), at every part of the brain’s white matter. MD measures the extent to which water is able to diffuse in any direction (so is high in areas such as the cerebro-spinal fluid, where its motion is relatively unconstrained), whereas FA measures what proportion of that diffusion is constrained (so is high in areas such as white matter, where myelinated axonal walls mean water can diffuse easily along, but not across, cells). These measures have been extensively applied to schizophrenia datasets and have almost invariably shown patients to be different to healthy controls (Kanaan et al., 2005), with their FA usually lower and their MD usually higher, suggesting some disruption to the normal white matter architecture.

The biological interpretation of these differences is more difficult, however, with reduced myelination and disordered fibre orientation popular candidate explanations, (Beaulieu, 2002) with global or regional distributions arguably linked to a genetic origin (Voineskos, 2015). Functional interpretations are also common, with symptoms not only seen as manifested by differences in specific tracts but also as potential causes of those symptoms through their abnormal activity (Samartzis et al., 2014). The timing of differences may illuminate their origin, to a degree – a congenital origin would be supported by differences found in those at risk but not yet unwell, for example, whereas those occurring late in the illness may be more likely to be consequences of illness itself or its treatment (Kuswanto et al., 2012; Samartzis et al., 2014). Their distribution may also be informative – although changes will almost certainly be multifactorial in origin, local differences would point to contributions from the involvement of a specific symptom, for example, or of a local environmental impact (Zalesky et al., 2012), whereas global differences would suggest more distributed contributions, such as from genetic sources (Kochunov et al., 2010).

The overwhelming evidence to date is that whenever and wherever these white matter differences in schizophrenia are sought they are found, and every major tract has now been implicated (Kanaan et al., 2005, 2009). Whether the differences are found to be local or global appears to depend in large part on the method of analysis (Kanaan et al., 2006, 2009), and many of them, at least, can be demonstrated at the earliest stages of the illness (Kuswanto et al., 2012; Samartzis et al., 2014). To attempt an answer to the question of the timing and distribution of differences, a sample would need to cover a wide age range, whether cross-sectionally or longitudinally, and be able to isolate the contribution of the analytical approach to localisation.

In this study, we sought to investigate the extent and development of differences in DTI measures in patients with schizophrenia. In a previous voxel-based analysis (VBA) (Kanaan et al., 2009), we found these differences to be very widespread indeed, with no relationship of FA with age in patients, and here sought to extend that investigation, with a larger sample and two complementary analytical methods (voxel-based, and tract-based). Voxel-based methods look for clusters of difference anywhere in the brain, while tract-based approaches look for differences that conform to a particular white matter tract or tracts. Combining these should permit precise localisation of differences (with the tract-based approach), while avoiding the limitation, if they agree, that any result is dependent on a specific analysis method. We expected to confirm those earlier findings, hypothesising that differences would be found throughout white matter, and that FA would not be related to age in patients with schizophrenia.

## Methods

### Ethics statement

All subjects gave written, informed consent after the study was explained to them, and the study was approved by the local Research Ethics Committee.

### Subjects

A total of 106 patients meeting *Diagnostic and Statistical Manual of Mental Disorders* (4th ed.; DSM-IV) (American Psychiatric Association [APA], 1994) criteria for schizophrenia were recruited from the wards and outpatient clinics of the South London and Maudsley hospital National Health Service (NHS) Trust and by national referral. Diagnoses were established by an experienced psychiatrist, using semi-structured interview and detailed case-note review. Patients who were assessed within the first few months of their illness had the DSM 6-month duration criterion confirmed at subsequent follow-up. The median duration of illness (defined as time from first contact with services) was 8.4 years (range 3 months to 37 years); all but 11 patients were receiving antipsychotic medication at the time of scanning. A total of 205 healthy volunteers were then matched to them from a sample of over 400 healthy controls. After exclusions following visual inspection of scans (see section ‘Image acquisition and pre-processing’ below), the remaining 104 patients and 200 controls matched for age (mean [standard deviation, SD]: patients 32.7 [10.3]; controls 33.3 [12.6]; *t*-test *p* = 0.7), gender (patients 88 male, controls 155 male; chi-squared *p* = 0.2) and handedness (102 patients, 190 controls were right handed; Fisher’s exact *p* = 0.2). The groups also matched on ethnicity (62/9 Caucasian/non-Caucasian for patients, 87/13 for controls, Fisher’s exact *p* = 1) and parental social class (chi-squared *p* = 0.5), but not IQ (mean [SD] patients 103.8 [10.4]; controls 109.8 [11.2]; *t*-test *p* = <0.001; measured by the National Adult Reading Test [NART] [Nelson, 1991]), although there were extensive missing values for these measures (48 patients and 128 controls for parental social class; 18 patients and 26 controls for IQ). Control subjects were excluded if they had a personal history of mental illness or a family history of psychotic illness, and both patients and controls were excluded if there was a lifetime history of head injury with loss of consciousness, neurological disease or drug/alcohol dependence. The sample included 144 subjects previously studied (Kanaan et al., 2009).

### Image acquisition and pre-processing

Diffusion-weighted imaging data were acquired using a GE Signa 1.5 Tesla LX magnetic resonance imaging (MRI) system (General Electric, Milwaukee, Wisconsin, USA) with a standard birdcage quadrature head coil, using an echo planar imaging sequence peripherally gated to the cardiac cycle and optimised for the acquisition of white matter DTI. Seven non-diffusion-weighted images (*b* = 0) were acquired, along with 64 images with diffusion gradients (*b* = 1300 s/mm^2^) uniformly distributed in space at each of 60 slices. The TR was 15 cardiac R-R intervals with a TE of 107 ms. Whole-head acquisition gave isotropic (2.5 mm^3^) voxels, reconstructed to a 1.875 × 1.875 mm in-plane pixel size. See Jones et al. (2002) for full details. Following mutual-information image correction (diffusion images individually registered to the mean image – see Catani et al., 2002), in-house software was used to remove non-brain tissue, determine the diffusion tensor using multivariate linear regression on log-transformed signal intensities, and calculate the FA and MD in each remaining voxel (Basser et al., 1994). Scans were manually inspected before further processing, and seven excluded (two patients and five controls – one patient and one control for grossly enlarged ventricles, the other five for image quality).

### VBA

To allow VBA, the FA and MD scans were first normalised using a two-stage process: a study-specific template was first created, and then the FA and MD images registered to it. To create the study-specific template, the mean *b* = 0 image from every subject was registered using SPM2 (Wellcome Department of Imaging Neuroscience, London, UK) to the SPM2 EPI template. The derived mapping parameters for each subject were then applied to that subject’s FA image. These normalised FA images were themselves averaged, and smoothed with an 8-mm Gaussian kernel. The FA images were then registered to this new template, again using SPM2, and the registration parameters applied to the MD. The registered FA images were segmented (using the default tissue probability information – ‘priors’ – in SPM2), and these probabilistic maps thresholded at 10% probability to generate liberal white matter masks. The registered FA and MD images were then smoothed with a 5-mm kernel, before masking to create white-matter-only FA and MD maps.

The statistical analyses were voxel-based analyses of variance (ANOVAs) of these white matter FA and MD maps of patients vs controls. This was carried out in XBAM_v4 (Institute of Psychiatry, London), employing a permutation-based method. The one-way ANOVA was fitted to each voxel of the normalised, segmented FA (or, separately, MD) maps, using patient status as the grouping variable. The ANOVA was only fitted at voxels where all subjects contributed; when combined with the liberal thresholding described above, this confined analysis to the body of the white matter. After fitting the ANOVA model to the observed data, the subject labels were randomly permuted between the two groups to achieve the null hypothesis of no main effect of group membership on FA. This permutation was carried out 1000 times at each voxel to allow the construction of a voxel-level null distribution of FA differences. After determination of those voxels showing significant effects at a set threshold (*p* < 0.05), sets of spatially contiguous supra-threshold voxels were identified, and the sum of the supra-threshold voxel-wise test statistics, or ‘mass’, of each three-dimensional cluster calculated. The mass of each cluster was then tested against the corresponding permutation distribution, and cluster-wise probability-thresholds chosen to ensure less than one false positive in the imaging volume.

### Tract-based spatial statistics

To permit tract-specific exploration of differences, between-group FA and MD comparisons were also conducted using TBSS version 1.2 (Smith et al., 2006). FA images from all participants were aligned to the Johns Hopkins University–International Consortium of Brain Mapping DTI-81 white matter atlas (JHU DTI atlas) (Mori et al., 2008) using FMRIB’s non-linear image registration tool (FNIRT) in FSL (http://fsl.fmrib.ox.ac.uk/fsl/fslwiki/). The mean of the voxel-wise FA images was ‘skeletonised’ (to generate a study-specific mean FA ‘skeleton’ representing the centres of tracts common to all participants) and thresholded for white matter (FA > 0.3). The aligned maps were then projected onto the mean white matter skeleton, and then subdivided according to the 48 regions of the JHU DTI atlas, with FA averaged per region per subject, and these regional means compared between groups using repeated-measures ANOVA with follow-up t-tests, and effect sizes (Cohen’s d), calculated with IBM SPSS v22 (www.ibm.com/software/analytics/spss). Correlations between FA and age were sought, and alternative models (quadratic, cubic, exponential, logarithmic, exponential, logistic) of the relationship also explored using SPSS, using R-squared as a measure of model-fit. Finally, Randomise (v2.1) in FSL was used to investigate group differences in FA across the skeletonised whole-brain maps. The analysis used threshold-free cluster enhancement (TFCE) (Smith and Nichols, 2009), considering 5000 permutations per contrast to generate voxel-wise probability values corrected for multiple testing. The registration and projections derived from this process were then applied to the MD images, and the statistical analyses repeated on them.

## Results

### VBA

**Figure 1.**
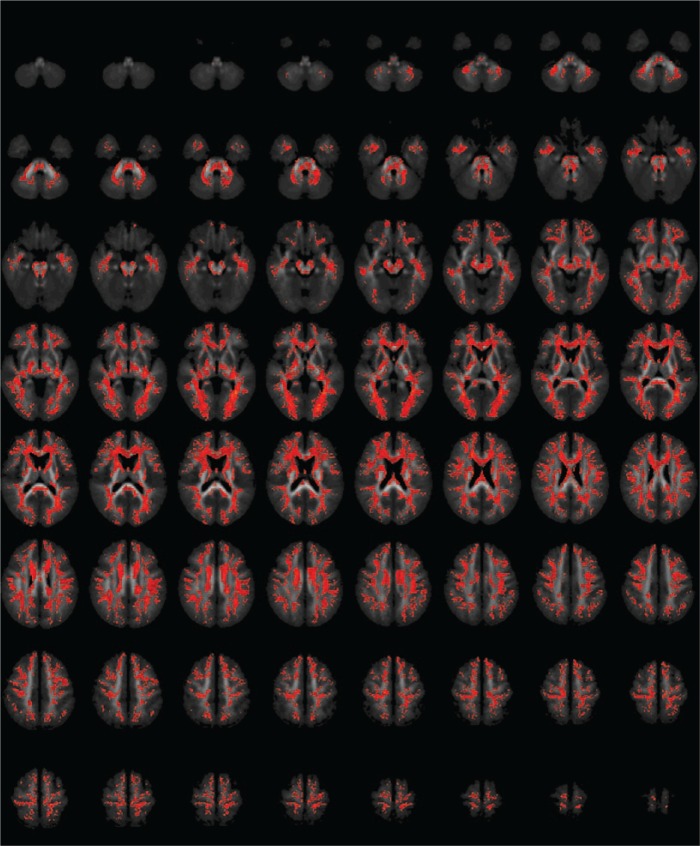
Areas of significantly decreased fractional anisotropy in patients with schizophrenia compared with healthy controls. The figure shows axial slices through the brain. Areas in red indicate decreased FA in patients. Voxel threshold *p* = 0.05, cluster threshold *p* = 0.001, corrected to less than one false-positive cluster.

At a voxel-wise threshold of 0.05, both FA and MD required cluster-wise thresholds of 0.001 to ensure less than one false positive. For FA, this resulted in a single huge cluster, covering most of the brain, with its ‘centre of mass’ in the forceps major of the corpus callosum, where FA was lower in patients than controls; for MD, it resulted in a slightly smaller, but still very large, cluster, with its centre in the body of the corpus callosum, where MD was higher in patients than controls. There were no clusters of higher FA or lower MD in patients. Details are shown in Table 1 and Figures 1 (FA) and 2 (MD).

**Table 1.** Clusters of reduced fractional anisotropy and increased mean diffusivity in patients compared to controls. Size is in voxels; Tal(x, y and z), refer to Talairach coordinates of centre of cluster; region denotes tract where centre of cluster lies.

Index	Size	Tal(x)	Tal(y)	Tal(z)	Region	*p*-value
Fractional anisotropy	35,444	−25.28	−57.41	6.00	Corpus Callosum	0.000001
Mean diffusivity	18,930	5.42	−18.52	18.00	Corpus Callosum	0.000002

**Figure 2.**
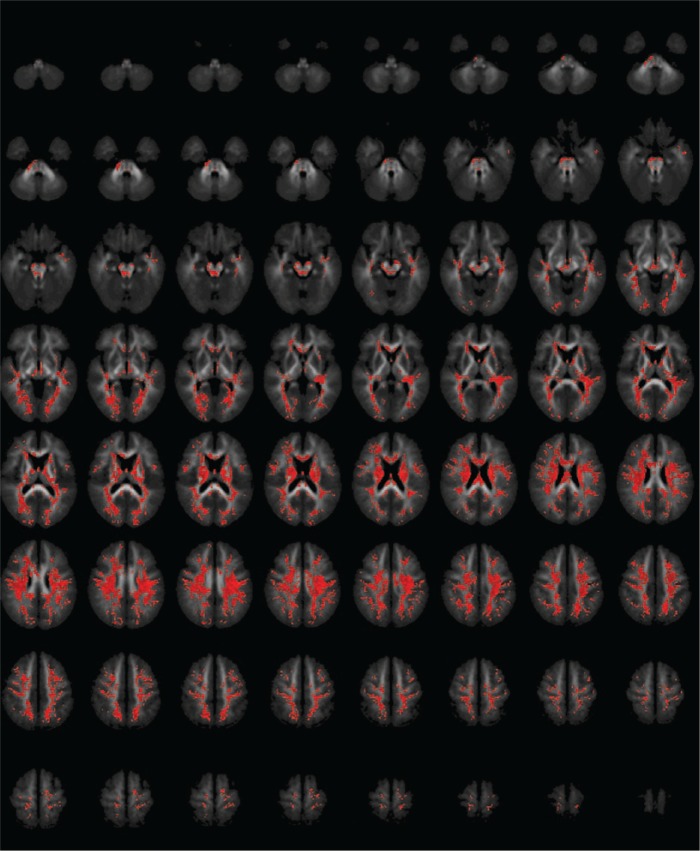
Areas of significantly increased mean diffusivity in patients with schizophrenia compared with healthy controls. The figure shows axial slices through the brain. Areas in red indicate increased MD in patients. Voxel threshold *p* = 0.05, cluster threshold *p* = 0.001, corrected to less than one false-positive cluster.

### TBSS analysis

**Figure 3.**
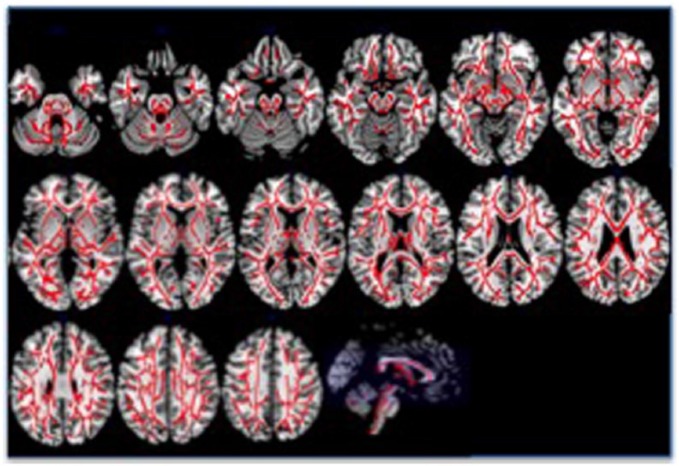
Skeletonised white matter areas where fractional anisotropy is decreased in patients with schizophrenia compared with healthy controls. The figure shows axial slices through the brain, followed by a sagittal section with each axial slice marked in blue. Numbers in blue reflect the Talairach z-coordinate. Areas in red indicate decreased FA in patients within the white matter, projected onto a white matter skeleton. Results are corrected for multiple comparisons using threshold-free cluster enhancement.

**Figure 4.**
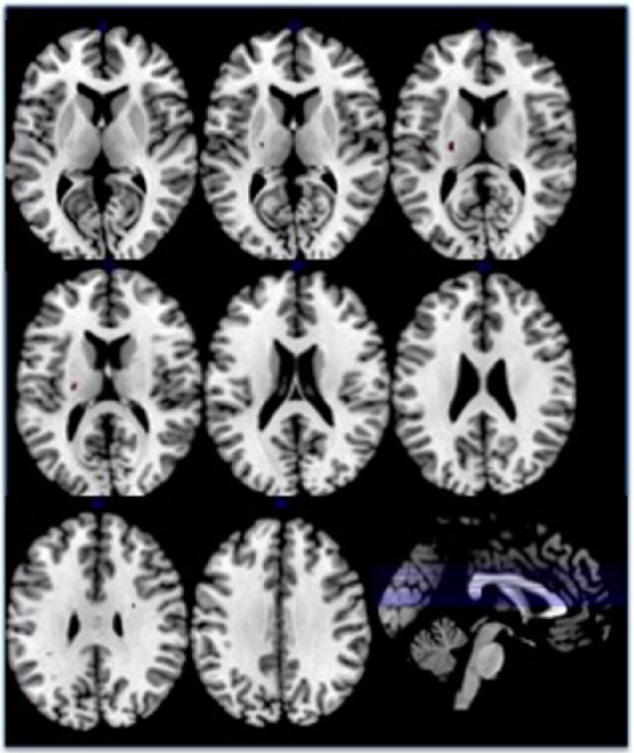
Skeletonised white matter areas where fractional anisotropy is increased in patients with schizophrenia compared with healthy controls. The figure shows axial slices through the brain, followed by a sagittal section with each axial slice marked in blue. Numbers in blue reflect the Talairach z-coordinate. Areas in red indicate increased FA in patients within the white matter, projected onto a white matter skeleton. Results are corrected for multiple comparisons using threshold-free cluster enhancement.

The TFCE analysis of the whole of the skeletonised white matter showed extensive reductions in FA in patients compared with controls, again covering virtually all of white matter (Figure 3); there were, however, two very small areas of increased FA in patients – one in the left internal capsule, and one in the area of the right superior corona radiata (Figure 4). The repeated-measures ANOVA, with Tract as within-subject and Group as between-subjects factor, found main effects of Tract (*p* < 0.001) and Group (*p* < 0.001), and a Tract × Group interaction (*p* = 0.019; Greenhouse–Geisser corrected). These effects were all still significant when IQ was included as a covariate (Tract and Group *p* < 0.001, Tract × Group *p* = 0.017; Greenhouse–Geisser corrected). Post hoc *t*-tests showed that all tracts had lower FA in patients than controls, except the Tapetum, which showed only a trend, with effect sizes varying from small (0.31 for the right uncinate fasciculus) to medium (0.78 for the left posterior thalamic radiation), representing 3–5% lower FA in the patient group – see Table 2. Note the *t*-tests are reported uncorrected for multiple comparisons, but correcting using the False Discovery Rate (Benjamini and Hochberg, 1995) did not change the significance of any of the results; Bonferroni correction would still leave the majority of tract differences significant (32 of 48), and these are indicated in the table.

**Table 2.** Fractional anisotropy differences in 48 discrete tracts between patients with schizophrenia and healthy controls.

Tract name	Mean (SD) FA patients (*n* = 104)	Mean (SD) FA controls (*n* = 200)	Effect size	*p*-value
Anterior corona radiata L	0.463 (0.034)	0.480 (0.031)	0.53	<0.001*
Anterior corona radiata R	0.469 (0.036)	0.490 (0.030)	0.65	<0.001*
Anterior limb internal capsule L	0.555 (0.038)	0.572 (0.027)	0.55	<0.001*
Anterior limb internal capsule R	0.572 (0.040)	0.591 (0.027)	0.59	<0.001*
Body corpus callosum	0.659 (0.064)	0.679 (0.040)	0.40	0.004
Cerebral peduncle L	0.667 (0.038)	0.684 (0.025)	0.57	<0.001*
Cerebral peduncle R	0.680 (0.041)	0.697 (0.027)	0.52	<0.001*
Cingulum/hippocampus L	0.508 (0.054)	0.526 (0.040)	0.40	0.003
Cingulum/hippocampus R	0.517 (0.054)	0.535 (0.042)	0.39	0.003
Cingulum L	0.592 (0.055)	0.610 (0.035)	0.42	0.003
Cingulum R	0.563 (0.051)	0.579 (0.035)	0.39	0.004
Corticospinal tract L	0.563 (0.046)	0.576 (0.032)	0.35	0.014
Corticospinal tract R	0.554 (0.041)	0.566 (0.032)	0.34	0.004
External capsule L	0.452 (0.032)	0.467 (0.025)	0.54	<0.001*
External capsule R	0.462 (0.030)	0.476 (0.024)	0.53	<0.001*
Fornix	0.437 (0.075)	0.476 (0.068)	0.55	<0.001*
Fornix/stria terminalis L	0.529 (0.043)	0.551 (0.030)	0.63	<0.001*
Fornix/stria terminalis R	0.528 (0.044)	0.546 (0.035)	0.47	<0.001*
Genu corpus callosum	0.688 (0.050)	0.705 (0.034)	0.42	0.002
Inferior cerebellar peduncles L	0.533 (0.048)	0.549 (0.030)	0.43	0.003
Inferior cerebellar peduncles R	0.531 (0.047)	0.549 (0.029)	0.50	<0.001*
Medial lemniscus L	0.558 (0.042)	0.576 (0.029)	0.53	<0.001*
Medial lemniscus R	0.564 (0.044)	0.586 (0.030)	0.62	<0.001*
Middle cerebellar peduncles	0.540 (0.036)	0.554 (0.022)	0.51	<0.001*
Pontine crossing tracts	0.481 (0.037)	0.496 (0.027)	0.49	<0.001*
Posterior corona radiata L	0.477 (0.035)	0.493 (0.026)	0.54	<0.001*
Posterior corona radiata R	0.490 (0.034)	0.504 (0.026)	0.48	<0.001*
Posterior limb internal capsule L	0.660 (0.033)	0.670 (0.023)	0.37	0.007
Posterior limb internal capsule R	0.652 (0.031)	0.664 (0.022)	0.47	0.001*
Posterior thalamic radiations L	0.584 (0.045)	0.612 (0.030)	0.78	<0.001*
Posterior thalamic radiations R	0.578 (0.041)	0.603 (0.030)	0.73	<0.001*
Retrolenticular internal capsule L	0.579 (0.037)	0.596 (0.025)	0.57	<0.001*
Retrolenticular internal capsule R	0.568 (0.033)	0.580 (0.026)	0.42	0.001*
Sagittal stratum/ILF L	0.522 (0.038)	0.543 (0.031)	0.63	<0.001*
Sagittal stratum/ILF R	0.532 (0.037)	0.551 (0.029)	0.59	<0.001*
Splenium corpus callosum	0.745 (0.041)	0.759 (0.022)	0.47	0.002
Superior cerebellar peduncles L	0.611 (0.056)	0.632 (0.037)	0.47	0.001*
Superior cerebellar peduncles R	0.601 (0.054)	0.623 (0.036)	0.51	<0.001*
Superior corona radiata L	0.493 (0.033)	0.510 (0.028)	0.57	<0.001*
Superior corona radiata R	0.485 (0.031)	0.500 (0.026)	0.54	<0.001*
Superior FOF L	0.474 (0.040)	0.488 (0.033)	0.39	0.001*
Superior FOF R	0.476 (0.045)	0.490 (0.034)	0.37	0.004
Superior longitudinal fasciculus L	0.515 (0.037)	0.532 (0.026)	0.56	<0.001*
Superior longitudinal fasciculus R	0.512 (0.038)	0.529 (0.027)	0.55	<0.001*
Tapetum L	0.594 (0.080)	0.609 (0.064)	0.21	0.096
Tapetum R	0.530 (0.076)	0.543 (0.057)	0.20	0.096
Uncinate L	0.464 (0.050)	0.479 (0.045)	0.32	0.010
Uncinate R	0.504 (0.053)	0.519 (0.047)	0.31	0.010

FA: fractional anisotropy; L: Left; R: Right; ILF: inferior longitudinal fasciculus; FOF: fronto-occipital fasciculus; SD: standard deviation.

* Significant at 0.05 after Bonferroni correction.

The MD effects were generally less pronounced than those for FA, with the only significant differences being greater MD in the patients in the fornix, right posterior thalamic radiations, left superior corona radiata and trends to greater MD in the patients’ right superior longitudinal fasciculus and pontine crossing tracts; there were no tracts with significantly lower MD in patients, and no trends thereto. The repeated-measures ANOVA found a main effect of tract (*p* < 0.001), and a tract by group interaction (*p* < 0.001, Greenhouse–Geisser corrected), but no main effect of group.

### Relationship with age

**Figure 5.**
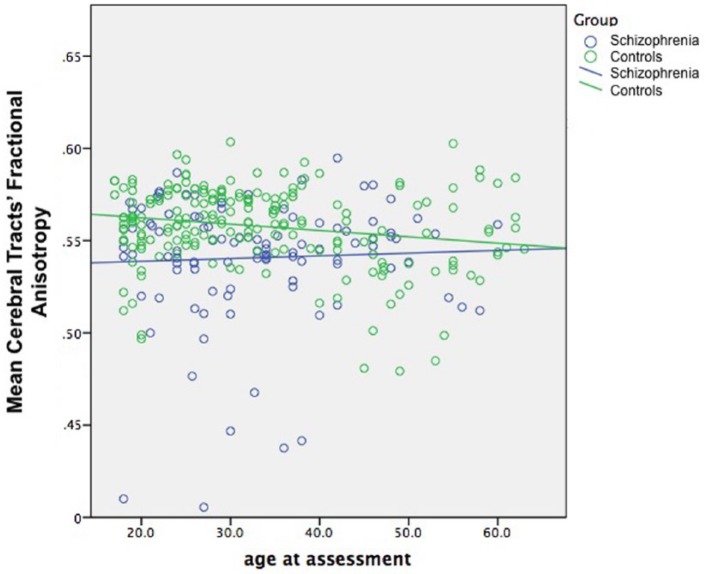
Scatterplot of mean cerebral fractional anisotropy in patients with schizophrenia and healthy controls. Patients *r* = 0.045; controls *r* = −1.198; *p* = 0.044.

An ANOVA on the mean of the TBSS cerebral tracts’ FA for each subject, with group and age as between-subjects factors, found a main effect of group (*F* = 8.5; *p* = 0.004), a trend to an effect of age (*p* = 0.068), with an age × group interaction (*p* < 0.001). Including IQ as a covariate did not change any of the significances. Examining post hoc correlations: in healthy controls, FA significantly correlated negatively with age in most tracts, although a few showed no significant correlation, and the bilateral superior cerebellar peduncles correlated positively with age, as previously noted (Kanaan et al., 2016); the patients’ tracts showed correlations of FA with age that were in most cases numerically smaller (closer to 0), but in no case were these differences from controls statistically significant (by *t*-tests on Fisher’s R to Z transformed correlations; Table 3). However, taking the mean of the cerebral tracts’ FA, the correlation with age did differ between groups (patients *r* = 0.045; controls *r* = −0.198; *p* = 0.044, two-tailed *t*-test on R-Z transforms; see Figure 5), and shows a larger difference in younger people, reducing with age, with the healthy controls FA declining to match that of the patients. The exploration of alternative models for the FA–age relationship in the cingulum, the fornix and the mean of the cerebral tracts found no model fitted better than linear.

**Table 3.** Fractional anisotropy correlations with age in patients with schizophrenia and healthy controls.

Tract name	Correlation of FA with age in patients	Correlation of FA with age in controls
Anterior corona radiata L	−0.098	−0.319**
Anterior corona radiata R	−0.093	−0.333**
Anterior limb internal capsule L	0.039	0.039
Anterior limb internal capsule R	0.088	0.020
Body corpus callosum	0.055	−0.161*
Cerebral peduncle L	−0.032	−0.281**
Cerebral peduncle R	−0.008	−0.286**
Cingulum/hippocampus L	0.136	−0.022
Cingulum/hippocampus R	0.147	0.019
Cingulum L	0.132	−0.121
Cingulum R	0.058	−0.055
Corticospinal tract L	−0.022	−0.051
Corticospinal tract R	−0.115	−0.090
External capsule L	0.093	−0.098
External capsule R	0.108	−0.144*
Fornix	−0.292**	−0.451**
Fornix/stria terminalis L	0.042	−0.192**
Fornix/stria terminalis R	−0.006	−0.224**
Genu corpus callosum	−0.046	−0.151*
Inferior cerebellar peduncles L	0.027	−0.144*
Inferior cerebellar peduncles R	0.087	−0.122
Medial lemniscus L	0.082	0.089
Medial lemniscus R	0.047	0.118
Middle cerebellar peduncles	0.072	−0.034
Pontine crossing tracts	−0.070	−0.010
Posterior corona radiata L	0.087	−0.306**
Posterior corona radiata R	0.043	−0.259**
Posterior limb internal capsule L	−0.006	−0.214**
Posterior limb internal capsule R	−0.035	−0.236**
Posterior thalamic radiations L	0.003	−0.269**
Posterior thalamic radiations R	0.048	−0.236**
Retrolenticular internal capsule L	0.057	−0.236**
Retrolenticular internal capsule R	−0.039	−0.247**
Sagittal stratum/ILF L	0.119	−0.025
Sagittal stratum/ILF R	0.041	−0.078
Splenium corpus callosum	0.073	−0.051
Superior cerebellar peduncles L	0.272**	0.390**
Superior cerebellar peduncles R	0.235*	0.364**
Superior corona radiata L	−0.154	−0.268**
Superior corona radiata R	−0.181	−0.334**
Superior FOF L	0.025	0.027
Superior FOF R	−0.055	−0.004
Superior longitudinal fasciculus L	0.112	−0.174*
Superior longitudinal fasciculus R	0.079	−0.151*
Tapetum L	0.188	0.023
Tapetum R	0.134	−0.028
Uncinate L	0.154	0.014
Uncinate R	0.179	0.120

FA: fractional anisotropy; L: Left; R: Right; ILF: inferior longitudinal fasciculus; FOF: fronto-occipital fasciculus.

* Significant at 0.05, uncorrected; **significant at 0.001, uncorrected.

The MD effects in the ANOVA were again less clear. Of those tracts where a significant difference in mean MD was found, the fornix found clear effects of group (*p* = 0.004) and age (*p* < 0.001), but only a trend towards a group × age interaction (*p* = 0.085); the right posterior thalamic radiations found no main or interaction effects; and the left superior corona radiata found a main effect of age (*p* < 0.001), and an interaction with group (*p* = 0.036), but no main effect of group.

## Discussion

These results indicate that patients with schizophrenia have lower FA throughout the brain’s white matter – although there is regional (tract) variation – and that MD is extensively raised as well. There were even two small areas of increased FA; however, these were only present in one of the three analyses (TFCE, but not VBA or the tract-based analysis): this makes them more likely to be artefactual, although of course were they real, they would confirm there is at least some local variation in these effects. While there are many influences on FA, including axon diameter and packing, membrane permeability, the ‘partial volume’ effect of adjacent structures and the crossing of tracts within a voxel of measurement (Jones et al., 2013), the two greatest influences are normally neuronal coherence and myelination (Beaulieu, 2002). While we cannot know from these data which combination of factors has contributed to the reduced FA – for changes in all have been reported in schizophrenia (Kanaan et al., 2005) – the co-location of increased MD (increased diffusion of water overall) suggests reduced myelination is more important, and reduced neuronal coherence less so. Explanations such as partial volume effect or the influence of crossing fibres, although their local influence may be profound, seem unlikely to account for such widespread differences.

The results also suggest this difference in FA narrows with age. While these are cross-sectional data, healthy controls show a small negative correlation with age, globally and in most tracts, suggestive of the gentle decline that has been widely reported (Glahn et al., 2013; Kochunov et al., 2009, 2012; Lebel and Beaulieu, 2011). In contrast, patients showed a very slight positive association with age, at least globally, with patients’ FA approaching control levels in older subjects. While the FA difference itself therefore does not appear to be progressive, the biological interpretation of this would be speculative, particularly as the biological causes of the decline in healthy ageing are unclear, probably multifactorial, and regionally variable (Bennett et al., 2010; Burzynska et al., 2010). Still, the result suggests either that these normal processes do not have the same impact on patients with schizophrenia (perhaps because they have already occurred) – or they are balanced by other processes to yield no net decline. These results have been found before, albeit in much smaller samples (Jones et al., 2006; Voineskos et al., 2010) – and conflict with those reporting accelerated age-related decline in schizophrenia (Friedman et al., 2008; Kochunov et al., 2013), again in smaller samples. Although there will be many possible explanations for this division within the field, differences in analysis method and sampling seem likely candidates. Although no study is immune from these influences, our sample was comparatively large, and we used a combination of analytical approaches covering all of white matter. Our sample will inevitably be limited in its generalisablity, however, notably by the tertiary-referral of some of the subjects, and, like the rest of the field to date, it is cross-sectional in nature, so inferences to changes will always require confirmation from longitudinal studies.

One potential confound is the age distribution of FA in schizophrenia. The relationship of FA and age across the lifespan is clearly not linear in the healthy: it increases with myelination, peaking around adolescence, and then declines throughout adulthood. The timing of the peak varies with the tract, with the cingulum earlier and the fornix later, for example (Lebel and Beaulieu, 2011). Therefore, it is possible that what looks like a comparatively flat age–FA relationship in schizophrenia is actually a delayed, flatter maturational peak, as has been suggested (Karlsgodt et al., 2012). Alternatively, it could be that maturation occurs so much earlier in schizophrenia that by adulthood, its decline is largely complete. These questions cannot be fully addressed with these data, although we tested for alternative models and found none to fit better than a linear one – and we note that our sample, which is an adult one, is much more likely to approximate a linear fit than one which includes adolescents, and thus the peak.

Other interpretations will be more speculative. A flatter age relationship does not support an explanation in terms of antipsychotic damage, at least not an ongoing one (Garver et al., 2008). The widespread distribution, combined with the lack of progression, if that is indeed what these results show, suggests the causes are present early, and relatively fixed. This would make global genetic causes more attractive explanations, although the local expression of these will vary (Romme et al., 2016), and the functional adaptation to symptoms or local environment less so, although of course a combination always remains possible.
